# The clinical features and prognosis of infective endocarditis in the elderly from 2007 to 2016 in a tertiary hospital in China

**DOI:** 10.1186/s12879-019-4546-6

**Published:** 2019-11-06

**Authors:** Zhenzhu Wu, Yi Chen, Tingting Xiao, Tianshui Niu, Qingyi Shi, Yonghong Xiao

**Affiliations:** State Key Laboratory for Diagnosis and Treatment of Infectious Diseases, National Clinical Research Center for Infectious Diseases, Collaborative Innovation Center for Diagnosis and Treatment of Infectious Diseases, The First Affiliated Hospital, College of Medicine, Zhejiang University, Hangzhou, China

**Keywords:** Infective endocarditis, Older patients, Risk factors, Surgical treatment

## Abstract

**Background:**

Infective endocarditis (IE) especially in the elderly is a serious disease, with a worse prognosis.

**Methods:**

A retrospective cohort study was conducted. A total of 405 patients with definite IE were divided into three groups: 205 patients under 50 years old, 141 patients between 50 and 64 years old and 59 patients over 65 years old.

**Results:**

For older patients, clinical symptoms such as fever, anemia, and heart murmur were as common as the younger patients. IE in old patients had more frequent nosocomial origin (*P* = 0.007) and tended to be more frequent with bad oral hygiene (*p* = 0.008). The most frequent isolated pathogens in the old groups was *streptococci* and *coagulase-negative staphylococci.* The old patients had a lower operation rate (40.7% vs 58.9% vs 62.4%, *P* = 0.012) and higher in-hospital mortality (20.3% vs 10.6% vs 8.8%, *P* = 0.044) compared with the younger patients. Surgical treatment was a significant predictor of one-year mortality even after adjusting for the confounders (HR = 2.45, 95% CI 1.027–10.598, *P* = 0.009). The one-year survival rate was higher for older patients with surgical intervention than those without (95.8% vs 68.6%, *P* = 0.007).

**Conclusions:**

Older patients with IE presented with more comorbidities, bad oral hygiene, more nosocomial origin and a more severe prognosis than younger patients. *Streptococci* was the most frequent micro-organisms in this group. Surgery were underused in old patients and those with surgical treatment had better prognosis.

## Background

Infective endocarditis (IE) is a severe disease with a high burden of mortality and morbidity [1]. Over the past few decades, with the increase in life span and invasive procedures, IE has become more and more frequent in the elderly [2, 3]. The increasing age of patients with IE will become the major determinant of disease characteristics in the future. Therefore, it is significantly important to explore the clinical features of old patients with IE at present.

According to the published researches, IE in the elderly is a different disease, with a higher mortality [2, 3]. The most frequent causative organism as reported previously is *Staphylococcus aureus* and *methicillin-resistant Staphylococcus aureus* has a high infection rate in older patients [2, 3]. Previous reports show that clinical presentations of IE in the elderly are often nonspecific and atypical, which often lead to the delay in diagnosis and treatment for this unique population [4–6]. And the proportion of patients undergoing surgical treatment is lower in older patients compared with the younger because of the increased risk caused by aging [7].

However, most of the reports derive from developed countries, and studies exploring the clinical features of older patients with IE in developing countries are scarce. The aim of this article is to investigate the clinical features and prognosis of IE in the elderly from a tertiary teaching hospital in east China.

## Methods

### Patient selection and study design

The study was conducted in the First Affiliated Hospital of Zhejiang University, Hangzhou, China. Patients with definite IE from January 1, 2007 to December 31, 2016 were reviewed. All patients were identified according to the modified Duke criteria [8] and patients under 16 years old were excluded. Patients with IE who were admitted to the hospital more than once during the study period were considered as one case. All the patients included were separated into three groups according to their age: 205 patients under 50 years old, 141 patients between 50 and 64 years old and 59 patients over 65 years old. Then, patients over 65 years old were divided into the survival group and the mortality group according to their one-year outcome.

The analysis strategies were as the follows: (1) To analyze the clinical features of IE in the elderly, a comparison between the three groups was conducted; (2) to explore the prognostic risk factors of one-year mortality for older patients and a comparison between the survival group and mortality group was conducted; (3) to evaluate the effect of surgical treatment on older patients, and analysis of the one-year survival rate between patients with antibiotic therapy combined with surgical intervention and patients with antibiotic treatment alone.

### Clinical parameters and definition

The data was obtained from the Electronic Medical Record. The following data were recorded: age, gender, underlying diseases general, IV drug addiction, dental condition and interventions, length of hospitalization, clinical, laboratory and microbiological data, vegetation location, complications of IE, treatments (included antibiotics and surgical intervention), and outcomes.

The modified Duke criteria, which were used to evaluate our patients, involve two major criteria: (1) the presence of at least two positive blood cultures with typical organisms consistent with IE, and (2) evidence of endocardial involvement, primarily diagnosed using echocardiography; and several minor criteria such as predisposing cardiac condition or injection drug abuse, fever >38°C, vascular phenomena (arterial embolism, septic pulmonary infarction, intracranial hemorrhage, Janeway lesions) or immunologic phenomena (Osler nodes, Roth spots, glomerulonephritis), and serological evidence of organisms consistent with IE. To be enrolled in this study as definite IE, patients had to meet one of the following criteria: (1) the major two criteria; (2) one major and three minor criteria; (3) five minor criteria; or (4) pathological criteria: microorganisms demonstrated by culture or on histological examination of a vegetation, a vegetation that has embolized, or an intracardiac abscess specimen; or pathological lesions; vegetation or intracardiac abscess by histological examination showing active endocarditishis.

Surgical indications were based on the European Society of Cardiology (ECS) guidelines: heart failure, uncontrolled infection and prevention of embolism were the main indications of surgery [8, 9].

The main outcome was one-year all-cause mortality. The one-year follow-up data were collected from the patients’ latest visits to our hospital. Transthoracic echocardiogram was performed routinely in all patients. Transesophageal echocardiogram (TEE) was used to detect cases with negative transthoracic echocardiography (TTE) results. Blood culture was performed in all the patients with aerobic, anaerobic and fungal blood cultures, but blood cultures for the HACEK group (*Haemophilus spp, Aggregatibacter spp, Cardiobaterium hominis, Eikenella corrodens, and Kingella kingae*), and anti-legionella, mycoplasma and bartonella anti-body tests as well as PCR test were not performed when patients had negative blood culture results.

### Statistical analysis

The clinical features analysis was performed using Pearson’ s χ2 test or Fisher’ s exact test as appropriate for categorical variables and independent Student’s t-test or Rank sum test was used as appropriate for continuous variables. Cox univariate and multivariate survival analysis was performed to discover the predictors of one-year all-cause mortality. A Kaplan-Meier analysis was used to determine the one-year survival. All tests were 2-tailed, and *P* < 0.05 was considered statistically significant. All analyses were performed using the SPSS version 23 statistical software.

## Results

### Patient enrollment

Between January 2007 and December 2016, there were 405 patients (male: 64.4%, age: 16–86) included in our study. Of these patients, 205 patients were in the < 50 years group, 141 patients in the 50–64 years group and 59 patients in the ≥65 years group. During the follow-up, 92.8% (378 of 407) of patients taking part in the study completed a median follow-up of one year after infective endocarditis diagnosis.

### The clinical characteristics of IE in the three groups

The clinical characteristics of IE in the three groups are summarized in Table 1.

**Table 1:** Clinical characteristics of patients with infective endocarditis

Variables % or Mean ±SD	<50 years old *n* = 205 (50.6%)	50-64 years old *n* = 141 (34.8%)	≥65 years old *n* = 59 (14.6%)	*P* value
Clinical data				
Male	127 (62.0)	93 (66.0)	41 (69.5)	.508
Age	35 ± 9	57 ± 4	72 ± 5	*P*<0.001
Hospital stay	24 ±19	28 ±27	23 ±19	.295
Duration of symptoms before echocardiography	30 ± 37	24 ± 27	24 ± 34	.174
Duration of symptoms before diagnosis	42 ± 39	42 ± 44	42 ± 58	.991
Time interval from diagnosis to surgery	23.5 (8–55.5)	16 (6.5–54)	30 (11.5–64)	.322
Time interval from embolism to diagnosis	4 ± 3	13 ± 17	11 ± 10	.001
Time interval from cerebral embolism to diagnosis	4 ± 2	16 ± 21	13 ± 12	.006*
IE localization				
Native valve	191 (93.2)	128 (90.8)	49 (83.1)	.059
Mitral	61 (30.0)	44 (31.2)	15 (25.4)	
Aortic	54 (26.3)	56 (40.0)	18 (30.5)	
Mitro-aortic	31 (15.1)	17 (12.1)	8 (13.5)	
Right valves	23 (11.2)	2 (1.4)	4 (6.8)	
Prosthetic valve	13 (6.3)	10 (7.1)	8 (13.6)	.176
Mitral	4 (2.0)	4 (2.8)	3 (5.1)	
Aortic	5 (2.4)	4 (2.8)	3 (5.1)	
Mitro-aortic	1 (0.5)	0	1 (1.7)	
Pacemaker	1 (0.5)	3 (2.1)	2 (3.4)	.191
Community origin	187 (91.2)	124 (87.9)	47 (79.7)	.050
Nosocomial origin	3 (1.5)	6 (4.3)	6 (10.2)	.007
Comorbidities				
Predisposing cardiac conditions				
Rheumatic heart disease	28 (13.7)	30 (21.3)	11 (18.6)	.169
Congenital heart disease	71 (34.6)	23 (16.3)	10 (16.9)	*P*<0.001
Previous cardiac surgery	16 (7.8)	14 (9.9)	14 (23.7)	.002
Degenerative heart disease	1 (0.5)	6 (4.3)	6 (10.2)	.001
Chronic pulmonary disease	1 (0.5)	3 (2.1)	2 (3.4)	.191
History of cancer	3 (1.5)	6 (4.3)	2 (3.4)	.275
Hemodialysis	3 (1.5)	7 (5.0)	4 (6.8)	.069
Liver cirrhosis	2 (1.0)	1 (0.7)	1 (1.7)	.813
Hypertension	14 (6.8)	43 (30.5)	25 (42.4)	*P*<0.001
Diabetes	2 (1.0)	25 (17.7)	10 (16.9)	*P*<0.001
Intravenous drug abuse	2 (1.0)	0	1 (1.7)	.250
Immunodepression	17 (8.3)	12 (8.5)	4 (6.8)	.915
Bad oral hygiene	57 (27.8)	45 (31.9)	29 (49.2)	.008
Symptoms and signs				
Fever	186 (90.7)	125 (88.7)	53 (89.8)	.820
Anemia	116 (56.6)	68 (48.2)	38 (64.4)	.085
Osler nodule	5 (2.4)	1 (0.7)	1 (1.7)	.439
Janeway lesions or nailbed bleeding	3 (1.5)	1 (0.7)	0	.601
New or changing heart murmur	172 (83.9)	110 (78.0)	45 (76.3)	.253
Hepatomegaly	10 (4.9)	9 (6.4)	2 (3.4)	.658
Splenomegaly	68 (33.2)	28 (19.9)	13 (22.0)	.015
Echocardiographic data				
Vegetation length				.029
<10 mm	35 (17.1)	36 (25.5)	18 (30.5)	
≥ 10 mm	146 (71.2)	83 (58.9)	30 (50.8)	
No vegetation	24 (11.7)	22 (15.6)	11 (18.6)	
Vegetation mobility	84 (41.0)	47 (33.3)	26 (44.1)	.238
Moderate or severe valve regurgitation	29 (14.2)	17 (12.4)	12 (20.3)	.547
Moderate or severe valve stenosis	11 (5.4)	27 (19.7)	5 (8.5)	.001
Abscess	25 (12.2)	17 (12.1)	8 (13.6)	.953
Annular abscess	13 (6.4)	10 (7.1)	7 (11.9)	.365
Pseudoaneurysm	18 (8.8)	7 (5.0)	2 (3.4)	.207
Valvular perforation	32 (15.8)	27 (19.1)	13 (22.0)	.480
Microbiology				
Streptococci	53 (25.9)	34 (24.1)	13 (22.0)	.819
Streptococcus viridans	22 (10.7)	9 (6.4)	3 (5.1)	.219
Staphylococci	40 (19.5)	28 (19.9)	11 (18.6)	.981
*Staphylococcus aureus*	17 (8.3)	9 (6.4)	2 (3.4)	.405
Coagulase-negative staphylococci	23 (11.2)	19 (13.5)	9 (15.3)	.660
Enterococci	2 (1.0)	2 (1.4)	1 (1.7)	
Fungi	0	2 (1.4)	1 (1.7)	
Polymicrobial	1 (0.5)	1 (0.7)	0	
Complications				
Heart failure	101 (49.3)	76 (53.9)	37 (62.7)	.181
Total emboli	57 (27.8)	45 (31.9)	23 (39.0)	.247
Emboli under treatment	60 (29.3)	35 (24.8)	12 (20.3)	.339
Intracranial infection	12 (5.9)	4 (2.8)	1 (1.7)	.227
Cerebral emboli	31 (15.1)	29 (20.6)	14 (23.7)	.219
Cerebral hemorrhage,	14 (6.8)	7 (5.0)	2 (3.4)	.544
Arrhythmia	24 (11.7)	33 (23.4)	23 (39.0)	*P*<0.001
Atrial fibrillation	15 (7.3)	29 (20.6)	20 (33.9)	*P*<0.001
Apparition of atrioventricular block	4 (2.0)	4 (2.8)	4 (6.8)	.155
Hepatic insufficiency	43 (21.0)	22 (15.6)	6 (10.2)	.119
Renal insufficiency	34 (16.6)	29 (20.6)	18 (30.5)	.061
Surgery and mortality				
Surgical indication	204 (99.5)	140 (99.3)	57 (96.6)	.142
Surgery indicated and performed	128 (62.4)	83 (58.9)	24 (40.7)	.012
Reason of no surgery				
Medical treatment	61 (80.3)	46 (80.7)	23 (69.7)	
Death before surgery	12 (15.8)	11 (19.3)	10 (30.3)	.216
Patient’s refusal	3 (3.9)	0	0	
In-hospital mortality	16 (7.8)	15 (10.6)	12 (20.3)	.023**
One-year mortality	18 (8.8)	15 (10.6)	12 (20.3)	.044
Cause of death				.124
Heart failure	0	5 (33.3)	3 (25.0)	
Sepsis	10 (55.6)	6 (40.0)	5 (41.7)	
Cerebral hemorrhage Brain palsy	6 (33.3)	1 (6.7)	2 (16.7)	
Life-threatening arrhythmias	1 (5.6)	1 (6.7)	1 (8.3)	
Others	1 (5.6)	2 (13.3)	1 (8.3)	

*IQR* interquartile range, *IE* Infective endocarditis

*p = 0.051: < 50 years group vs ≥65 years group *p* = 0.526:50–64 years group vs ≥65 years group

** *p* = 0.006 < 50 years group vs ≥65 years group *p* = 0.067 50–64 years group vs ≥65 years group

There was no statistically significant difference between the age groups in terms of the length of hospital stay, duration of symptoms before echocardiography, duration of symptoms before diagnosis, time interval from diagnosis to surgery and time interval from cerebral embolism to diagnosis. Prosthetic valve IE was more common in older patients and native valve IE was more common in younger patients but there were no statistically difference. Old patients with IE had more frequent nosocomial origin (*P* = 0.007).

It was more common for old IE patients to have previous cardiac surgery (*P* = 0.001) and degenerative heart disease (P = 0.001) but less common in congenital heart disease (P<0.001). There was no statistically significant difference between the age groups in terms of fever, anemia, heart murmur, extracardiac IE signs (Osler’s nodes, Janeway lesions and nailbed bleeding) and hepatomegaly. However, splenomegaly was less frequent in old patients. Old patients tend to be more frequent with a bad oral hygiene (*p* = 0.008).

The most frequent isolated pathogens in the old groups was *streptococci* and *coagulase-negative staphylococci*. *Staphylococcus aureus* tended to be less frequent with age but it presented no statistically difference. Concerning complications, no difference was observed between groups in heart failure, emboli under treatment, intracranial infection, cerebral emboli, cerebral hemorrhage, hepatic insufficiency and renal insufficiency, except atrial fibrillation, which was more frequent in old subjects.

In-hospital mortality rates were 7.8% (< 50 years group), 10.6% (50–65 years group) and 15.7% (≥65 years group) (*p* = 0.023). In-hospital mortality was high in the ≥65 years group than in the< 50 years group (*p* = 0.006). In the ≥65 years group, the predominant cause of deaths were sepsis (41.7%) and heart failure (25.0%).

According to the ESC guidelines, surgery was theoretically indicated in 99.5, 99.3 and 96.6%, but was ultimately performed in 62.4,58.9 and 40.7% in the < 50 years, 50–64 years and ≥ 65 years groups, respectively. The main reason for elder patients not operated was the choice of medical treatment after considering the high comorbidities, high operative risk or multidisciplinary decision. Owing to death before surgery tended to be more frequent with age (15.8 to 19.3% and 30.3%) but there was no statistically difference.

### The risk factors for one-year mortality in old patients

**Fig. 1:**
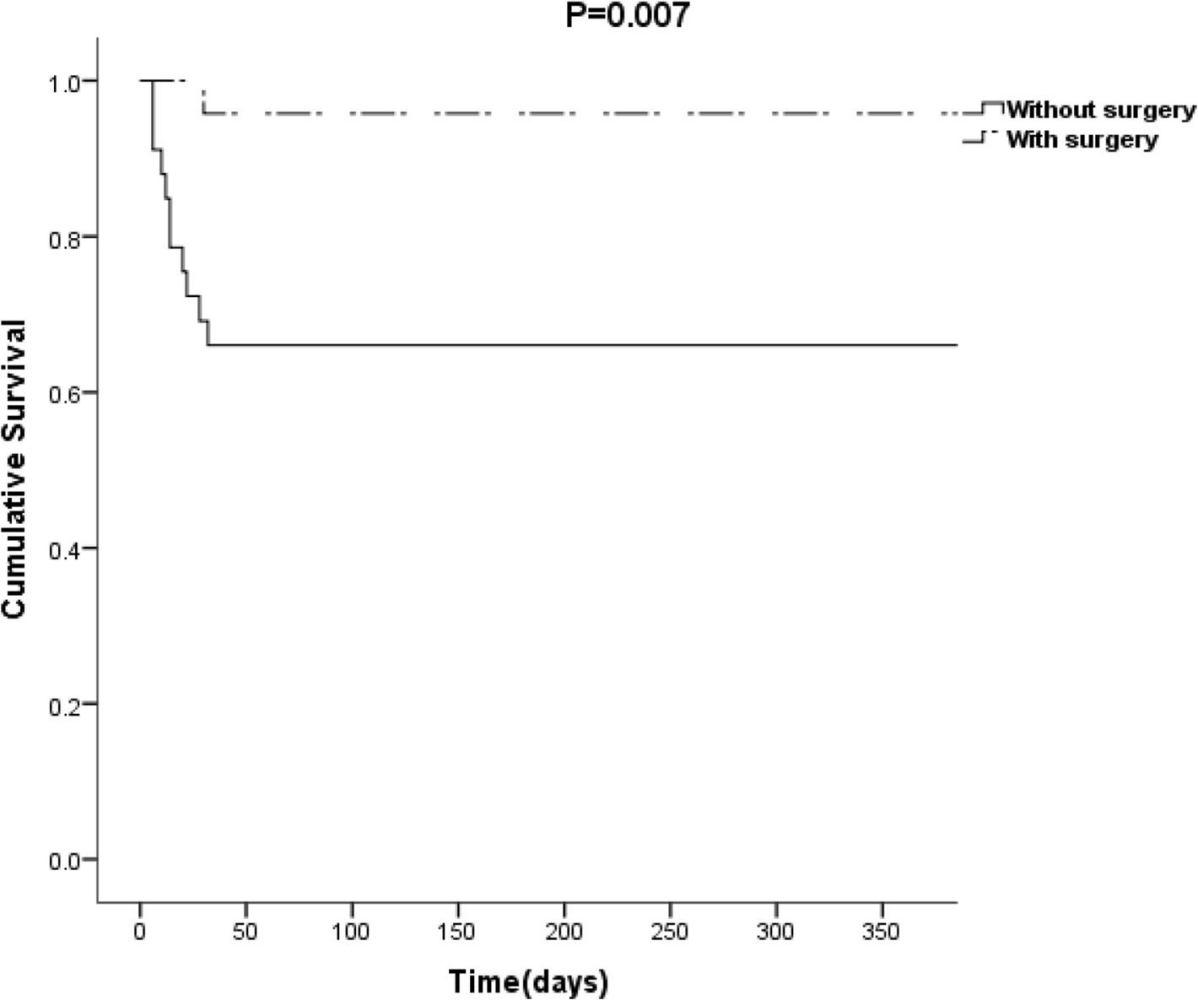
Figure One-year survival analysis for patients≥65 years old The Kaplan–Meier survival curves revealed that cumulative one-year survival rate was higher in old patients with surgical intervention than that in patients without surgical intervention (95.8% vs 68.6%, *P* = 0.007)

A one-year cox survival analysis was performed for the ≥65 years group. The results were show in Tables 2 and 3. Significant variables included man, hemodialysis, renal insufficiency, pulmonary arterial hypertension, Pitt score ≥ 4, vegetation length>30 mm and surgical treatment were risk factors for one-year mortality. Surgical treatment [HR = 2.45, 95% CI 1.027–10.598, *P* = 0.009) was a significant predictor of one-year mortality even after adjusting for confounder. The Kaplan–Meier survival curves revealed that cumulative one-year survival rate was significantly higher in old patients when surgery operated than those not (95.8% vs 68.6%, *P* = 0.007) (Fig. 1).

**Table 2:** Cox univariate analysis of one-year mortality in patients ≥65 year old with infective endocarditis

Variables% or Mean ±SD	Survival *n* = 47	Mortality *n* = 12	*P* value
Clinical data			
Male	35 (74.5)	6 (50.0)	.267
Length of hospital stay	25 ± 21	16 ± 10	.052
Symptoms before echocardiography	26 ± 37	16 ± 17	.355
Duration of symptoms before diagnosis, median (IQR),days	24.0 (12.0–42.0)	29.0 (10.3–54.8)	.799
Time interval from emboli to diagnosis	12 ± 10	11 ± 11	.868
Time interval from cerebral emboli to diagnosis	15 ± 13	12 ± 12	.724
IE localization			
Native valve	40 (85.1)	9 (75.0)	.518
Prosthetic valve	6 (12.8)	2 (16.7)	.862
Community origin	42 (89.4)	5 (41.7)	*P*<0.001
Nosocomial origin	2 (4.3)	4 (33.3)	*P*<0.001
Comorbidities			
With predisposing cardiac disease	29 (61.7)	6 (50.0)	.457
Rheumatic heart disease	8 (17.0)	3 (25.0)	.752
Congenital heart disease	9 (19.1)	1 (8.3)	.482
Previous cardiac surgery	10 (21.3)	4 (33.3)	.475
Degenerative heart disease	6 (12.8)	0	.466
Chronic pulmonary disease	1 (2.1)	1 (8.3)	.343
Cancer	2 (4.3)	0	.690
Hemodialysis	1 (2.1)	3 (25.0)	.007
Liver cirrhosis			.713
Hypertension	18 (38.3)	7 (58.3)	.272
Diabetes	7 (14.9)	3 (25.0)	.565
Immunodepression	0	4 (33.3)	*P*<0.001
Bad oral hygiene	22 (46.8)	7 (58.3)	.713
Symptoms and signs			
Anemia	31 (66.0)	7 (58.3)	.425
Fever	44 (93.6)	9 (75.0)	.046
Splenomegaly	11 (23.4)	2 (16.7)	.491
Hepatomegaly	1 (2.1)	1 (8.3)	.516
New or changing heart murmur	39 (83.0)	6 (50.0)	.021
Complications			
Heart failure	25 (53.2)	12 (100.0)	.106
Total emboli	16 (34.0)	7 (58.3)	.143
Emboli under treatment	8 (17.0)	4 (33.3)	.295
Intracranial infection	1 (2.1)	0	.713
Cerebral emboli	8 (17.0)	6 (50.0)	.053
Cerebral hemorrhage	1 (2.1)	1 (8.3)	.404
Arrhythmia	16 (34.0)	7 (58.3)	.202
Renal insufficiency	9 (19.1)	9 (75.0)	.001
Hepatic insufficiency	3 (6.4)	3 (25.0)	.092
Pulmonary arterial hypertension	15 (31.9)	8 (66.7)	.037
Moderate or severe valve regurgitation	10 (21.3)	2 (16.7)	.570
Moderate or severe valve stenosis	4 (8.5)	1 (8.3)	.912
Annular abscess	5 (10.6)	2 (16.7)	.564
Pseudoaneurysm	1 (2.1)	1 (8.3)	.289
Valvular perforation	10 (21.3)	3 (25.0)	.781
Pitt score ≥ 4	1 (2.1)	5 (41.7)	.001
Vegetation length			.062
≤10 mm	25 (53.2)	4 (33.3)	
>10 mm<20 mm	19 (40.4)	5 (41.7)	
≥20 mm ≤ 30 mm	3 (6.4)	1 (8.3)	
>30 mm	0	2 (16.7)	.039
Microbiology			.050
Streptococci	12 (48.0)	1 (16.7)	.268
Staphylococci	8 (32.0)	3 (50.0)	.679
Staphylococcus aureus	0	2 (33.3)	.039
Enterococci	1 (4.0)	0	
Fungi	0	1 (16.7)	
Surgical treatment	23 (48.9)	1 (8.3)	.018

**Table 3:** Cox multivariate analysis of one-year mortality for patients ≥65 year old with infective endocarditis

Variables	*P* value	HR	95% CI
Male	.025	3.751	1.183–11.891
Hemodialysis	.007	6.146	1.633–23.124
Renal insufficiency	.001	8.684	2.327–32.407
Pulmonary arterial hypertension	.037	3.627	1.083–12.153
Pitt score ≥ 4	<.001	10.589	3.284–34.147
Vegetation length>30 mm	.009	10.600	1.796–62.569
Surgical treatment	.009	2.45	1.027–10.598

*IQR* interquartile range, *HR* hazard ratio, *CI* confidence interval

## Discussion

This study represents a large cohort of IE from a single center. It showed that older patients with IE had common clinical symptoms, more nosocomial origin, worse oral hygiene than younger patients. The most frequent isolated pathogens in the old groups was *Streptococci*. Moreover, they presented more comorbidities, more atrial fibrillation as well as more severe prognosis than younger patients. Surgical therapy was less performed in older patients although the theoretical indications for surgery was clear. Those with surgical therapy had better outcome.

### The clinical characteristics of older patients with infective endocarditis

According to the published researches, the clinical features in older patients were few and untypical, which often led to a delay in the diagnosis of IE [5, 10, 11]. While, Jean et al. [12] found older people had a more severe clinical status than younger patients, which lead to the early diagnosis. However, the clinical presentations in old patients was not significantly different as compared with the younger patients in our study. And the time to diagnosis was not significantly different compared with the younger patients.

In accordance with previous studies, clinical characteristics varied with aging [12–15]. Older patients were more frail, which often lead to more cardiovascular and general comorbidities and complications than younger patients. In our study, the older patients presented more predisposing factors (like previous cardiac surgery history, degenerative heart disease, hypertension, diabetes and so on) contrary to younger patients who frequently presented congenital heart disease. For IE patients, comorbidities and complications increased with ageing, just like the general population.

Different to the published researches, *streptococci* was the most frequent isolated pathogens in the old groups in our study. This might be owing to the large number of native valve IE and community-acquired IE in old patients. According to the published researches, *streptococci* was more prevalent among patients with a native valve and community-acquired IE [16]. What’s more, the bad oral hygiene among old patients might be another important reason. The microtrauma caused by these everyday activities (like oral hygiene habits) has been identified to induce *oral streptococcal* bacteraemia [17]. Therefore, a better control for individual oral hygiene and dental status for old patients was important in reducing *oral streptococcal* infective endocarditis.

In our study, we found the in-hospital mortality rate and one-year mortality rate in older patients was much higher than the younger patients, which was consistent with previous reports [3, 14, 15, 18, 19]. As reported previously, older adults were prone to require complex care needs and suffer from multiple comorbidities, which made them vulnerable to health-associated exposure and poor outcomes [14, 20–22]. Besides, the lower operative rate in older patients compared with the younger in our cohort may be another important reason for the higher mortality in older patients [7, 13, 14].

The in-hospital mortality and one-year mortality were lower in older patients in our study compared with previous studies [5, 14, 18]. Léopold Oliver et al. reported that one-year mortality was higher in the ≥80-year-old group (37.3%) than in the < 65-year-old group (13%) and the 65–80-year-old group (19.7%), indicating that the mortality rate increased with aging [13]. The few number of very old patients in our study (there were only 5 patients who were over 80 years old) may be an important reason. And a larger cohort for older IE patients was suggest in our region in the future.

### Surgical therapy and prognosis for patients≥65 year old with infective endocarditis

Previous studies reported that older age, renal failure, prosthetic valve endocarditis, neurological deficit, and cerebral emboli were independent risk factors for one-year mortality in older patients [4, 15]. In our study we found the independent risk factors for one-year mortality were man, hemodialysis, renal insufficiency, Pitt score ≥ 4,vegetation length>30 mm and surgical treatment. These events have been confirmed previously to be risk factors for mortality in IE patients [8, 23, 24].

We observed that elder patients with surgical therapy had a lower mortality rate compared with patients not operated during the one-year follow-up. Other recent reports also reached the same conclusion [13]. Surgery was performed less frequent in older patients in our study, although the rate of patients with theoretical indications of surgery was not significantly different compared with the younger. This phenomenon was frequently presented in previous reports [3, 25, 26]. The main consideration may be the increasing risks during the perioperative period owing to the decline in organ function and the presence of comorbidities associated with aging. These factors made the choice of surgical treatment for elderly patients more difficult.

But these considerations could not prevent the old patients with surgical indications from suitable treatments in-time. There are many frailty scores to assess the physical condition of older patients, and some scores showed good reliability in the assessment of mortality independently of age [20]. Some studies have recently proven the utility of these scores for the evaluation of IE-related stroke and prognosis evaluation before cardiac surgery [27]. Therefore, surgery is appropriate in selected old patients with IE. And we suggest a more global patient evaluation and cooperation among multiple specialists to improve IE management in older populations.

### Limitations

There are several limitations in our study. First, it was performed in a referral teaching hospital where most patients were transferred from other medical centers leading to long-term disease and negative blood culture results. Therefore, these results should not be generalized to other patient groups. Second, as a retrospective study, the long-term follow-up was not possible and 29 patients were lost during the one-year follow-up. Finally, the study covered a long period of time in order to keep the enough sample sizes. Changes in treatment regimens and causative organisms could affect the patient prognosis during this period. Therefore, a multiple-center prospective cohort studies conducted in our region was suggested.

## Conclusions

In conclusion, older patients with IE presents more comorbidities, bad oral hygiene, more nosocomial origin and a more severe prognosis than younger patients. *Streptococci* was the most frequent micro-organisms in this group. Surgery were underused in older patients and those with surgical treatment presented better long-term prognosis.

## Data Availability

The datasets used and analysed during the current study are available from the corresponding author on reasonable request.

## Abbreviations

CI: Confidence interval
HR: Hazard ratio
IE: Infective endocarditis
IQR: Interquartile range
OR: Odds ratios
SD: Standard deviation
TEE: Transesophageal echocardiography
TTE: Transthoracic echocardiography

## References

[1] Cahill TJ, Baddour LM, Habib G, Hoen B, Salaun E, Pettersson GB (2017). Challenges in infective endocarditis. J Am Coll Cardiol.

[2] K RWSaL (2010). Health care exposure and age in infective endocarditis-results of a contemporary population-based profile of 1536 patients. Eur Heart J.

[3] Forestier E, Fraisse T, Roubaud-Baudron C, Selton-Suty C, Pagani L (2016). Managing infective endocarditis in the elderly: new issues for an old disease. Clin Interv Aging.

[4] Gagliardi JP, Nettles RE, McCarty DE, Sanders LL, Corey GR, Sexton DJ (1998). Native valve infective endocarditis in elderly and younger adult patients: comparison of clinical features and outcomes with use of the Duke criteria and the Duke endocarditis database. Clin Infect Dis.

[5] Selton-Suty C, Hoen B, Grentzinger A, Houplon P, Maignan M, Juilliere Y, Danchin N, Canton P, Cherrier F (1997). Clinical and bacteriological characteristics of infective endocarditis in the elderly. Heart..

[6] Werner GS, Schulz R, Fuchs JB, Andreas S, Prange H, Ruschewski W, Kreuzer H (1996). Infective endocarditis in the elderly in the era of transesophageal echocardiography-clinical features and prognosis compared with younger patients. Am J Med.

[7] Durante-Mangoni E, Bradley S, Selton-Suty C, Tripodi MF, Barsic B, Bouza E, Cabell CH (2008). Current features of infective endocarditis in elderly patients. Arch Intern Med.

[8] Linhartova K, Benes J, Gregor P (2016). 2015 ESC guidelines for the management of infective endocarditis. Summary document prepared by the Czech Society of Cardiology. Cor Et Vasa.

[9] Pettersson GB, Coselli JS, Hussain ST, Griffin B, Blackstone EH, Gordon SM (2017). 2016 the American Association for Thoracic Surgery (AATS) consensus guidelines: surgical treatment of infective endocarditis: executive summary. J Thorac Cardiovasc Surg.

[10] Thomas P, Allal J, Bontoux D, Rossi F, Poupet JY, Petitalot JP (1984). Rheumatological manifestations of infective endocarditis. Ann Rheum Dis.

[11] Lopez-Wolf D, Vilacosta I, San Roman JA, Fernandez C, Sarria C, Lopez J (2011). Infective endocarditis in octogenarian patients. Rev Esp Cardiol.

[12] Remadi JP, Nadji G, Goissen T, Zomvuama NA, Sorel C, Tribouilloy C (2009). Infective endocarditis in elderly patients: clinical characteristics and outcome. Eur J Cardiothorac Surg.

[13] Oliver L, Lavoute C, Giorgi R, Salaun E, Hubert S, Casalta JP (2017). Infective endocarditis in octogenarians. Heart..

[14] Lopez J, Revilla A, Vilacosta I, Sevilla T, Villacorta E, Sarria C (2010). Age-dependent profile of left-sided infective endocarditis: a 3-center experience. Circulation..

[15] Di Salvo G, Thuny F, Rosenberg V, Pergola V, Belliard O, Derumeaux G (2003). Endocarditis in the elderly: clinical, echocardiographic, and prognostic features. Eur Heart J.

[16] Njuguna B, Gardner A, Karwa R, Delahaye F (2017). Infective endocarditis in low- and middle-income countries. Cardiol Clin.

[17] Tubiana S, Blotiere PO, Hoen B, Lesclous P, Millot S, Rudant J (2017). Dental procedures, antibiotic prophylaxis, and endocarditis among people with prosthetic heart valves: nationwide population based cohort and a case crossover study. BMJ..

[18] Netzer RO, Zollinger E, Seiler C, Cerny A (1999). Native valve infective endocarditis in elderly and younger adult patients: comparison of clinical features and outcomes with use of the Duke criteria. Clin Infect Dis.

[19] López-Wolf D, Vilacosta I, San Román JA, Fernández C, Sarriá C, López J (2011). Infective endocarditis in octogenarian patients. Rev Esp Cardiol (English Edition).

[20] Sundermann SH, Dademasch A, Seifert B, Rodriguez Cetina Biefer H, Emmert MY, Walther T (2014). Frailty is a predictor of short- and mid-term mortality after elective cardiac surgery independently of age. Interact Cardiovasc Thorac Surg.

[21] Fried LP, Tangen CM, Walston J, Newman AB, Hirsch C, Gottdiener J (2001). Frailty in older adults: evidence for a phenotype. J Gerontol A Biol Sci Med Sci.

[22] McMillan GJ, Hubbard RE (2012). Frailty in older inpatients: what physicians need to know. Qjm..

[23] Samol A, Kaese S, Bloch J, Gorlich D, Peters G, Waltenberger J (2015). Infective endocarditis on ICU: risk factors, outcome and long-term follow-up. Infection..

[24] Nucifora G, Badano LP, Viale P, Gianfagna P, Allocca G, Montanaro D (2007). Infective endocarditis in chronic haemodialysis patients: an increasing clinical challenge. Eur Heart J.

[25] Ramirez-Duque N, Garcia-Cabrera E, Ivanova-Georgieva R, Noureddine M, Lomas JM, Hidalgo-Tenorio C (2011). Surgical treatment for infective endocarditis in elderly patients. J Inf Secur.

[26] Taradin GG, Vatutin NT, Prendergast BD, Newton JD, Chaus EA, Smyrnova AS (2016). Infective endocarditis in the elderly: the current view of the problem. Ter Arkh.

[27] Murai R, Funakoshi S, Kaji S, Kitai T, Kim K, Koyama T (2017). Outcomes of early surgery for infective endocarditis with moderate cerebral complications. J Thorac Cardiovasc Surg.

